# First-line endocrine therapy for postmenopausal patients with hormone receptor-positive, HER2-negative metastatic breast cancer: a systematic review and meta-analysis

**DOI:** 10.1007/s12282-020-01054-7

**Published:** 2020-02-11

**Authors:** Tatsunori Shimoi, Yasuaki Sagara, Fumikata Hara, Tatsuya Toyama, Hiroji Iwata

**Affiliations:** 1 Department of Breast and Medical Oncology, National Cancer Center Hospital, 5-1-1 Tsukiji, Chuo-ku, Tokyo, 104-0045 Japan; 2 Department of Breast Surgical Oncology, Hakuaikai Social Cooperation, Sagara Hospital, 3-31 Matsubara-cho, Kagoshima, 892-0098 Japan; 3 Department of Breast Medical Oncology, The Cancer Institute Hospital of the Japanese Foundation for Cancer Research, 3-8-31 Ariake, Koto-ku, Tokyo, 135-8550 Japan; 4 Department of Breast Surgery, Nagoya City University Graduate School of Medical Sciences, 1 Kawasumi, Mizuho-cho, Mizuho-ku, Nagoya, 467-8601 Japan; 5 Department of Breast Oncology, Aichi Cancer Center, 1-1 Kanokoden, Chikusa-ku, Nagoya, 464-8681 Japan

**Keywords:** Breast cancer, Aromatase inhibitor, Fulvestrant, Cyclin-dependent kinase 4/6 inhibitor, Meta-analysis

## Abstract

**Background:**

In establishing the 2018 Breast Cancer Practice Guidelines of the Japan Breast Cancer Society, we explored the optimal first-line endocrine therapy for advanced postmenopausal hormone receptor-positive breast cancer.

**Methods:**

We performed a systematic review of relevant reports from randomized-controlled studies published prior to November 2016 found using medical journal search engines. The main outcomes which we evaluated were progression-free survival (PFS), objective response rate (ORR), disease control rate (CBR), and toxicity.

**Results:**

Four controlled trials comparing aromatase inhibitors (AI) and cyclin-dependent kinase (CDK)4/6 inhibitor combination therapy to AI monotherapy, and two controlled trials comparing anastrozole to fulvestrant 500 mg were analyzed. AI/CDK4/6 inhibitor combination therapy significantly improved PFS (Risk Ratio: 0.67, 95%CI 0.60–0.73), increased ORR (Risk Difference: 0.11, 95% CI 0.07–0.16), and increased CBR (Risk Difference: 0.11, 95% CI 0.07–0.15), compared with AI monotherapy. Patients who received this combination therapy had a higher grade ≥ 3 adverse event rate more than those who received AI monotherapy (Risk Difference: 43%, 95%CI: 0.39–0.47). Fulvestrant 500 mg alone significantly improved PFS (risk ratio: 0.85, 95%CI 0.72–0.98), but ORR and CBR were similar to those of anastrozole alone.

**Conclusion:**

In the first-line treatment for advanced postmenopausal hormone receptor-positive breast cancer, a combination therapy of CDK4/6 inhibitors and AI showed significant improvement of PFS, ORR, and CBR but with significant increased toxicities compared with AI alone. Fulvestrant 500 mg monotherapy significantly prolonged PFS compared with AI monotherapy. We must wait for the results of the studies with longer follow-up period.

## Introduction

Breast cancer is the most frequent cancer in women worldwide, with 2 million new cases diagnosed in 2018 [1]. Also in Japan, breast cancer is a leading cancer in women, with more than 76,000 cases diagnosed in 2014, accounting for 20% of all cancer cases in women. Additionally, breast cancer mortality accounts for 8.8% of all cancer deaths in women [2].

Breast cancer prognosis has been prolonged due to the development of treatments targeted to early breast cancer and metastatic breast cancer. However, metastatic or unresectable disease is hard to be cured and the prognosis is still limited. Breast cancer is divided into four subtypes based on hormone receptor (HR) and human epidermal receptor 2 (HER2) statuses. HR-positive breast cancer is the most common subtype, with a frequency of more than 70% worldwide, consistent with Japanese Breast Cancer Society registry data [3, 4].

For postmenopausal patients with HR-positive, HER2-negative metastatic or recurrent breast cancer without life-threatening visceral metastatic diseases [5], endocrine treatment is usually recommended over chemotherapy for systemic therapy to improve disease control and prolong survival, because it has similar efficacy with fewer side effects [6].

In particular, multiple randomized-controlled trials (RCTs) comparing aromatase inhibitors (AI) to tamoxifen alone had been conducted by 2008. Multiple meta-analyses of primary endocrine therapy for postmenopausal HR-positive metastatic/recurrent breast cancer have shown that AI is more effective than other endocrine therapies, such as tamoxifen, and are used as first-line therapy worldwide [7–9]. Since 2015, multiple RCTs have reported the efficacy of single-agent fulvestrant 500 mg or an AI plus cyclin-dependent kinase (CDK)4/6 inhibitor combination therapy compared to AI monotherapy.

Fulvestrant is a selective estrogen receptor downregulator that promotes degradation of the estrogen receptor and blocks its function in breast cancer cells. It is an effective endocrine treatment for women with hormone-sensitive advanced breast cancer.

CDKs are a large family of serine-threonine kinases that play an essential role in the regulation of cell cycle progression. A CDK4/6 inhibitor inhibits excessive phosphorylation of CDK4 or CDK6 retinoblastoma protein and blocks cell cycle progression from G_1_ to S phase. Recently, the efficacy of combination therapy with CDK4/6 inhibitors and endocrine therapy has been investigated in clinical trials.

In devising the new 2018 breast cancer guidelines in Japan, we conducted a systematic review and meta-analyses to investigate what treatment options are optimal in the first-line therapy for postmenopausal patients with hormone receptor-positive, HER2-negative metastatic breast cancer.

## Materials and methods

### Search methods for the identification of studies

We performed a systematic review of all RCTs that met the inclusion criteria. We searched PubMed, the Cochrane Central Register of Controlled Trials, and Ichushi-web using the following terms: “breast neoplasms”, “antineoplastic agents, hormonal”, “estrogen antagonists”, “gonadotropin-releasing hormone”, “aromatase inhibitors”, “receptor, ERBB-2”, “cyclin-dependent kinases”, “protein kinase inhibitors”, “positive”, and “postmenopause” and its synonyms. We applied no restrictions in terms of language. The search was limited to articles published between January 1969 and November 2016. We included prospective phase II and III RCTs and clinical practice guidelines from the American Society of Clinical Oncology (ASCO) [10]. We also manually searched for relevant studies.

### Data extraction and article selection

A trained librarian independently extracted relevant data and standardized it in an EXCEL spreadsheet. Two authors (TS and YS) cross-checked all data and selected the trials to perform for the systematic review. If there were any disagreements between the two authors, a third author (FH) was consulted to resolve them.

**Fig. 1:**
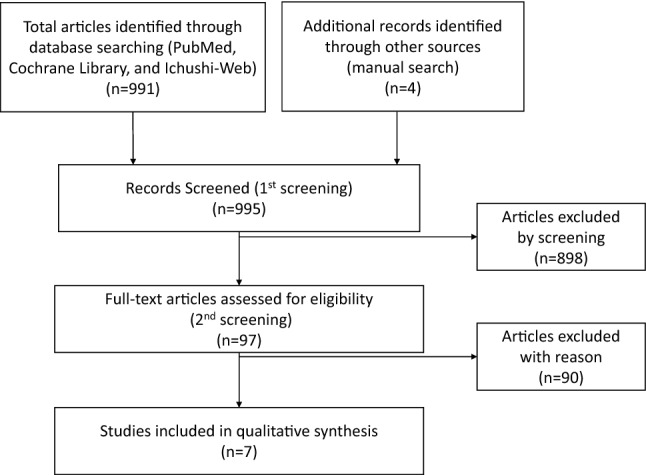
Flowchart summarizing the process of identifying eligible studies

Eligible trials for this analysis were those with the following characteristics: (1) RCTs that were published with publicly available data; (2) trials conducted in patients with locally advanced inoperable/metastatic HR-positive HER2-negative breast cancer; (3) studies comparing standard first-line endocrine therapies; (4) studies with available information on progression-free survival (PFS), time to progression (TTP), response, and adverse events; and (5) studies with sufficient information to estimate the risk ratio (RR) and 95% confidence interval (CI) for disease progression (Fig. 1)

The studies excluded from this analysis were those with the following characteristics: (1) randomized trials evaluating the efficacy of new endocrine strategies without containing first-line setting patients; (2) non-randomized studies; (3) phase I clinical trials; and (4) currently ongoing and/or unpublished studies from which insufficient results were available at the time of the literature search.

After data collection, if more than one RCT with the same endocrine therapies was found, each with a comparison between control and trial arms, we performed a meta-analysis or integrated analysis.

We evaluated the quality of the studies assessed in this process following the Minds Handbook for Clinical Practice Guideline Development 2014 [11].

The primary endpoint was PFS; the secondary endpoints were objective response (complete or partial) and clinical benefit rates (CBR; complete response, partial response, or stable disease over 24 weeks) in articles evaluated according to the RECIST version 1.1 guideline, and grade ≥ 3 adverse events.

We did not perform a new meta-analysis for fulvestrant plus anastrozole vs. AI or third-generation AI vs. tamoxifen, for which recent meta-analyses were found in the current literature search and an additional RCT could not be found for re-performing new meta-analyses [9, 12]. Comparative agents not subjected to a new RCT since the 2016 ASCO guidelines were excluded from the meta-analysis [10].

The Monarch 3 trial, in which the results of the major conference presentations were documented between November 2016 and the end of the guideline consensus meeting in December 2017, was added to the analysis.

### Statistical analysis

We used *I*^2^ to assess heterogeneity across studies. We estimated heterogeneity as low or moderate if *I*^2^ < 50%. When there was no statistically significant heterogeneity, we calculated the pooled effect with a random-effects model. We evaluated publication bias by visual inspection of funnel plots. We assessed the bias risk of individual studies with a review manager, version 5.3, from the Cochrane Collaboration. We considered a statistical test with *P* < 0.05 to be significant.

## Results

### Overview of the literature search

In total, we found 991 articles by electronic search. We further added four articles based on manual searches. After reviewing the titles and abstracts, we excluded 898 articles that did not meet our inclusion criteria or were duplicates. We reviewed the full text of 97 potentially eligible articles, and we excluded 90 articles, because there were no additional RCTs to allow us to perform meta-analyses. In total, we included seven articles (6 RCTs) for meta-analysis. Four were RCTs comparing AI plus CDK4/6 inhibitor to AI alone, and three were RCTs comparing fulvestrant to anastrozole (one article was the long-term follow-up of an RCT). All studies reported the median PFS/TTP, objective response rate (ORR), and adverse events.

### AI plus CDK4/6 inhibitor versus AI alone in first-line endocrine therapy

We performed a meta-analysis with four randomized phase III trials (PALOMA-1 trial, PALOMA-2 trial, MONALEESA-2 trial, Monarch 3 trial) that compared AI plus CDK4/6 inhibitor to AI monotherapy (Table 1) [13–16].

**Table 1:** Study Characteristics of trials investigating cyclin-dependent kinase 4/6 inhibitor combination therapy

First Author, year	Trial name	Arms	Number of patients	Median PFS (month)	Number of events			
					PFS (%)	ORR (%)	CBR (%)	AE G3 or more (%)
Finn 2015 [13]	PALOMA 1	PAL+LET^a^	84	20.2	41 (48.8)	36 (42.9)	68 (81.0)	63 (75.9)
		PLB+LET	81	10.2	59 (72.8)	27 (33.3)	47 (58.0)	16 (20.8)
Finn 2016 [14]	PALOMA 2	PAL+LET^a^	444	24.8	196 (44.1)	187 (42.1)	377 (84.9)	239 (53.8)
		PLB+LET	222	14.5	137 (61.7)	76 (34.2)	156 (70.2)	31 (14.0)
Hortobagyi 2016 [15]	MONALEESA 2	RIBO+LET^b^	334	Not reached	87 (26.1)	136 (40.7)	266 (79.6)	271 (81.1)
		PLB+LET	334	14.7	137 (43.7)	92 (27.5)	243 (72.8)	108 (32.3)
Goetz 2017 [16]	Monarch 3	Abe+NSAI^c^	328	Not reached	108 (32.9)	158 (48.2)	256 (78.1)	180 (54.9)
		PLB+NSAI	165	14.7	86 (52.2)	57 (34.6)	118 (71.5)	35 (21.2)

*PAL* palbociclib, *LET* letrozole, *PLB* placebo, *NSAI* nonsteroidal aromatase inhibitor

^a^Palbociclib (125mg daily for 21 d every 28 d) + letrozole (2.5mg daily) vs placebo + letrozole (2.5mg daily)

^b^Ribociclib (600mg daily for 21 d every 28 d) + letrozole (2.5mgdaily) vs placebo + letrozole (2.5mg daily)

^c^Abemaciclib (150mg twice daily every 28 d) + anastrozole (1mg daily) or letrozole (2.5mg daily) vs placebo + anastrozole (1mg daily) or letrozole (2.5mg daily)

**Fig. 2:**
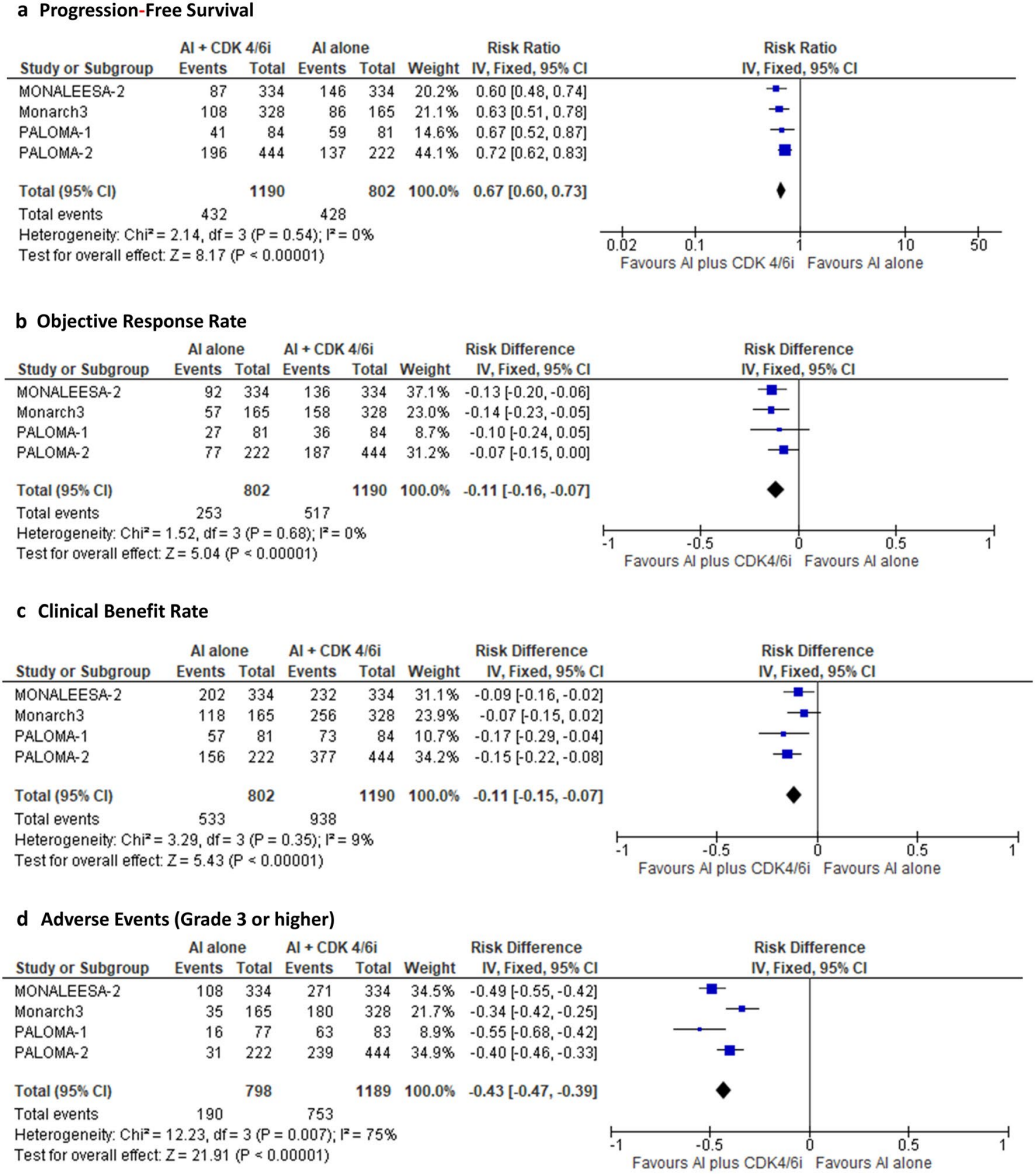
Meta-analysis of aromatase inhibitor, with or without concurrent use of cyclin-dependent kinase 4/6 inhibitor. **a** Progression-free survival, **b** overall response rate, **c** clinical benefit rate, and **d** adverse events (grade ≥ 3)

PFS, ORR, and CBR data were available in all four trials. This meta-analysis demonstrated that AI plus CDK4/6 inhibitor was associated with a improved PFS (RR, 0.67; 95% CI 0.60–0.73; *I*^2^ = 0%; *P* < 0.001) (Fig. 2a). Moreover, this meta-analysis demonstrated that AI plus CDK4/6 inhibitor was associated with an increased ORR [risk difference (RD), 0.11; 95% CI 0.07–0.16; *I*^2^ = 0%; *P* < 0.001] (Fig. 2b) and an increased CBR (risk difference, 0.11; 95% CI 0.07–0.15; *I*^2^ = 9%, *P* < 0.001) (Fig. 2c). The efficacy of the combination therapy of AI and CDK4/6 inhibitor was consistently higher than that of AI monotherapy. However, grade ≥ 3 adverse events were more frequent with combination therapy (RD, 0.43; 95% CI 0.39–0.47; *I*^2^ = 75%; *P* < 0.001) than with AI monotherapy, though the heterogeneity was high among studies (Fig. 2d) [13–16].

### Fulvestrant versus anastrozole in first-line endocrine therapy

**Fig. 3:**
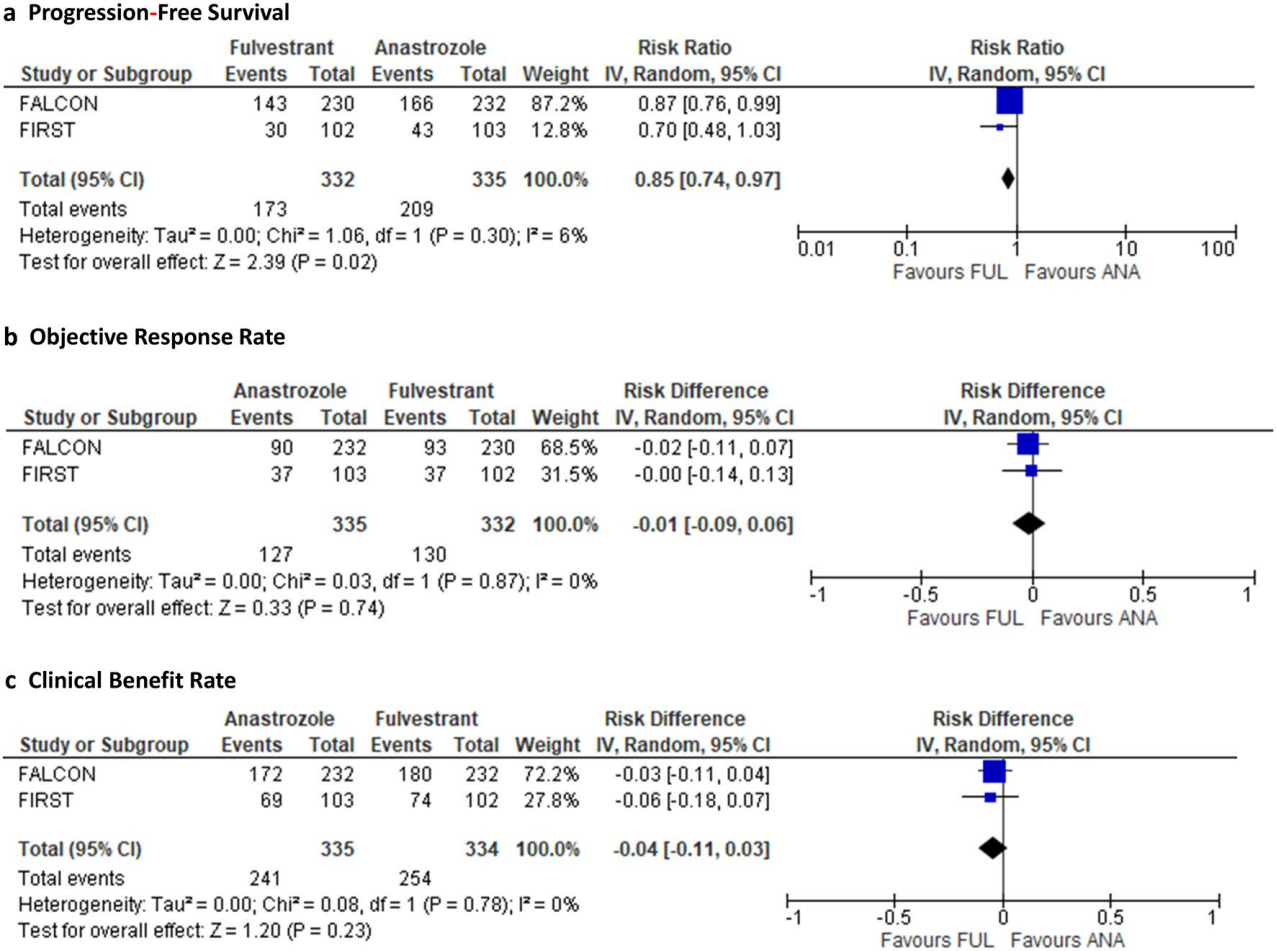
Integrated analysis comparing fulvestrant to anastrozole. **a** Progression-free survival, **b** overall response rate, and **c** clinical benefit rate

We found two randomized-controlled trials comparing fulvestrant 500 mg to anastrozole [17–19]. We performed an integrated analysis of the FIRST trial (a randomized phase II trial) and the FALCON trial (a phase III trial) (Table 2). As a result, PFS and TTP were prolonged with fulvestrant more than with anastrozole (RR, 0.84; 95% CI 0.72–0.98; *I*^2^ = 6%; *P* = 0.02) (Fig. 3) [17–19].

**Table 2:** Study characteristics of trials comparing fulvestrant 500 mg to anastrozole 1 mg

First Author, year	Trial name	Arms	Number of patients	Median PFS (month)	Number of events		
					PFS (%)	ORR (%)	CBR (%)
Robertson 2012 [18], Ellis 2015 [17]	FIRST	Fulvestrant 500 mg (days 0, 14, 28, and every 28 days thereafter)	102	23.4	30 (29.4)	32 (31.4)	74 (72.6)
		Anastrozole 1 mg daily	103	13.1	43 (41.8)	33 (32.0)	69 (67.0)
Robertson 2016 [19]	FALCON	Fulvestrant 500 mg (days 0, 14, 28, and every 28 days thereafter)	230	16.6	143 (62.2)	93 (40.4)	180 (78.3)
		Anastrozole 1 mg daily	232	13.8	166 (71.6)	90 (38.8)	172 (74.1)

The meta-analysis showed no significant different in ORR between fulvestrant and anastrozole (RD, 0.01; 95% CI − 0.06–0.09; *I*^2^ = 0%; *P* = 0.72). The meta-analysis also showed that there was no significant difference in CBR between fulvestrant and anastrozole (rate difference, 0.05; 95% CI − 0.02–0.11; *I*^2^ = 0%; *P* = 0.18). For the FIRST trial, we were unable to identify the frequency of grade ≥ 3 adverse events for each group and, therefore, could not perform an integrated analysis of adverse events.

### Examination of publication bias

The funnel plot showed no explicit publication biases (figure not shown).

## Discussion

In this study, we performed a systematic review and meta-analyses of the therapeutic effects and adverse events of both AI and CDK4/6 inhibitor combination therapy and fulvestrant 500 mg monotherapy compared to AI monotherapy as the optimal first-line endocrine therapy for postmenopausal women with HR-positive HER2-negative breast cancer. We compared AI and CDK4/6 inhibitor combination therapy with AI monotherapy based on the result of four clinical trials. Moreover, we compared fulvestrant 500 mg monotherapy with AI monotherapy based on three clinical trials.

Our analysis showed that the addition of a CDK4/6 inhibitor to AI was significantly associated with higher response rates, CBR, and prolonged PFS. The addition of a CDK4/6 inhibitor for combination therapy was also associated with severe grade ≥ 3 adverse events. Fulvestrant 500 mg monotherapy was associated with prolonged PFS compared to AI; however, no significant differences in ORR or CBR were observed.

The updated results of SWOG 0266 showed improvement in overall survival by combination therapy with anastrozole and AI, especially among patients without receiving previous endocrine therapy [20]. We did not include this finding, because our systematic review is restricted to the study published until 2016. The combination therapy would become an option among endocrine therapy naïve patient.

Our analysis has some limitations. First, at this time, no AI monotherapy conferred an improvement of overall survival when used for primary endocrine therapy, and the result of overall survival was not reported yet in these studies. Long-term outcome evaluations, including overall survival, have not been clarified for CDK4/6 inhibitor and AI combination therapy or fulvestrant 500 mg monotherapy as the first-line endocrine therapies. Therefore, clinicians must wait for the results of the studies with longer follow-up period. Second, adverse events have not been investigated in detail. Among the CDK4/6 inhibitors, palbociclib has a high frequency of neutropenia, whereas abemaciclib has a high frequency of diarrhea. When extrapolating data to patients, physicians should discuss treatment options, bearing in mind the different adverse event profiles of each drug. Finally, ribociclib has not applied for marketing approval in Japan; however, we included it in the meta-analysis. Because of the high efficacy and safety results among the study group as a whole, this does not seem to be a significant issue when applying our study into clinical practice in Japan.

As a clinically important parameter, prolonged PFS is already used as the primary endpoint in many clinical trials. For example, it is associated with delayed time to treatment with cytotoxic anticancer drugs, slowed the timing of toxicity, and reduced side effects and psychological burdens associated with anticancer drugs.

In conclusion, our meta-analysis showed that the combination of CDK4/6 inhibitors and AI significantly prolonged PFS and improved ORR and CBR compared to AI alone, but increased grade ≥ 3 adverse events. Moreover, our integrated analysis showed that fulvestrant 500 mg monotherapy significantly prolonged PFS compared to AI monotherapy, but there was no difference of ORR and CBR between fulvestrant and AI monotherapy.
